# 

**Published:** 1909-07-24

**Authors:** 


### BOOKS RECEIVED.

Shaw and Sons.

"Symptoms and their Interpretation," by James Mackenzie, M.D., M.R.C.P.

Baillière, Tindall, and Cox.

"The After-Treatment of Operations"—III. Edited by P. Lockhart Mummery, F.R.C.S.

Henry Kimpton.

"Practical Text-book of Midwifery"—IV. Edited by Robert Jardine, M.D., F.R.C.S., etc.

